# AN OUTBREAK OF COVID-19 INFECTION WITH NO MORTALITY IN A GERIATRIC INSTITUTION IN COLOMBIA

**DOI:** 10.1093/geroni/igad104.2262

**Published:** 2023-12-21

**Authors:** Carlos Reyes-Ortiz, Jose Ocampo-Chaparro

**Affiliations:** Florida A & M University, Tallahassee, Florida, United States; Universidad del Valle, Cali, Valle del Cauca, Colombia

## Abstract

Unusually, an outbreak of Covid-19 during the first year of the pandemic in an underserved geriatric institution had no mortality. This cross-sectional study aimed to determine factors associated with COVID-19 infection among 252 institutionalized older adults in Cali, Colombia’s larger geriatric hospital and nursing home, Geriatric Hospital and Nursing Home San Miguel assisting multiethnic, impoverished, and low-educated residents. This population had a median age of 80 years (range 55 to 103) and a median Barthel of 90.0; 50% were women, 19% had dementia, and 2% were obese. 84 (33.3%) were infected, and none died and did not go to the UCI or were referred to a higher-level hospital. In a multivariate logistic regression model, being infected was associated with age 80 and older, a lower Barthel score, and malnutrition. Due to collinearity, separated models showed an association between infection and higher scores on the clinical frailty scale or lower grip strength. All institutionalized older adults with Covid-19 infection in this geriatric institution survived. It was likely related to appropriate general medical and nursing management, including well-controlled comorbidities, and individualized interdisciplinary rehabilitation, attention, and support.

